# Three Needs and Information Anxiety on Knowledge Purchase Intentions across Online Knowledge Platforms

**DOI:** 10.3390/bs11100127

**Published:** 2021-09-22

**Authors:** Shinyi Lin, Kohang Cheng, Shu-Hui Chuang

**Affiliations:** 1 Master Program of Business Administration, Department of Creative Design and Management, National Taichung University of Education, Taichung 403, Taiwan; bbm107113@gm.ntcu.edu.tw; 2 Department of Business Administration, Asian University, Taichung 403, Taiwan; joyce@asia.edu.tw

**Keywords:** knowledge purchase intentions, online knowledge platforms, three needs, cognitive style, information anxiety

## Abstract

Given the recent advances in technology, knowledge-based products have become increasingly prevalent. Many companies offer interdisciplinary resources for incumbent learners to break through chronological and geographical constraints. Therefore, it is important to investigate the factors that motivate learners to pay for knowledge-based products. The purpose of this research was to identify the relevant factors that contribute to purchasing intentions and to clarify the reasons why people purchase knowledge-based products. This study involved 406 valid participants over 20 years of age with knowledge purchase experience. The results demonstrated that incumbent learners’ need for affiliation has a positive effect on involvement, and that involvement has a significant positive impact on knowledge purchase intentions. The key factor influencing learners to pay for knowledge-based products is their involvement in learning. Information anxiety interferes with the relationship between involvement and knowledge purchase intentions. However, no linear relationship was found between cognitive styles and involvement. Field-dependent learners show greater involvement and also information anxiety than field-independent learners. The research offers suggestions for practical use and future research from the perspective of knowledge-product marketing.

## 1. Introduction

According to the EBRD Knowledge Economy Index report by the European Bank for Reconstruction and Development [1], the continuously increasing knowledge economy (KE) is due to the rapid development of ICT infrastructure, which drives the progress of business services. Due to the increasing demand for knowledge content by learners, knowledge consumption behavior has shifted from a free-of-charge model to a payment model, where learners use knowledge payment platforms to gain interdisciplinary knowledge and skills [2,3]. That is, intangible knowledge has become a deliverable product through digital technology, and the value of these product depends on a learner’s cognitive ability.

Knowledge management (KM) aims to effectively facilitate the sharing and transmission of knowledge, in order to facilitate access [4]. An essential feature of knowledge management is properly preserving knowledge for future use through systematic planning [5]. Mardani, Nikoosokhan [6] describe three dimensions of knowledge management, including knowledge production, knowledge integration, and knowledge application, in which knowledge integration comprises knowledge storage and knowledge distribution (i.e., knowledge transfer). Therefore, knowledge has shifted from supply to demand [7]. The main process of knowledge transfer involves source-recipient communication, and depends on a person’s abilities, dispositions, or motivational factors [8]. As stated above, knowledge transfer involves two-way communication and is a dynamic process, thus knowledge demanders are typically proactive in their search for relevant information. Therefore, this study proposes that knowledge transfer and knowledge purchase intentions belong to the same concept. Upadhyay and Paul [9] argue that the relationship between learning and knowledge management is best understood through experiential learning.

According to an overall internet usage status survey conducted by the Taiwan Network Information Center [10], 85.6% of the Taiwanese population uses the internet daily, and there is little difference in the internet access rates of 12- to 54-year-olds. People primarily learn online by using online searches, and they believe that online learning is helpful to them. Furthermore, our survey found that computers are the most widely used online learning device. Based on the 2019 Digital Content Industry in Taiwan Report, there are three pillars of Taiwan’s digital content industry: Digital games, computer animation, and e-learning, of which e-learning is the most important, emphasizing educational services for knowledge transfer through innovative technologies. New knowledge payment platforms are continually increasing in prominence, with knowledge regarded as the main products sold on the platform. The main customer base is learners, i.e., people who want to self-educate or invest in themselves. In addition, our survey found that VoiceTube^TM^ Hero is the most commonly used knowledge-based services platform for Taiwanese users, and Hahow^TM^ is the second most commonly used platform. Hahow^TM^ provides the most diverse and interesting online courses for both educational advancement and career-oriented training. Through a unique course fundraising mechanism, individuals who enjoy self-learning and exchanging skills can efficiently complete online learning and independently gain achievements. Clearly, some people study a language as a goal, while others prefer interdisciplinary studies. The business model of knowledge payment platforms can be divided into two groups: A subscription model (i.e., through an annual fee or monthly fee) and outright purchasing habits (i.e., through a perpetual license). Learners can choose a suitable solution for themselves.

**Figure 1:**
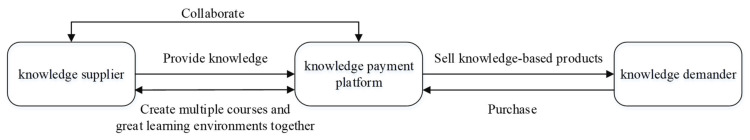
The business model of the online knowledge platform.

The three cores of knowledge payment are: The knowledge demander; knowledge supplier; and knowledge payment platform (Figure 1). Knowledge suppliers sell their professional knowledge to the platform as products, and knowledge demanders purchase the knowledge products they need. While, knowledge payment platforms represent a service broker, thereby creating multiple courses with knowledge suppliers and ensuring that the quality of the knowledge product conforms to learners’ preferences. Finally, knowledge payment platforms gain learners’ attention by offering multiple professional courses. At present, the main services of Taiwan’s knowledge payment platforms include two-way communications between lecturers and learners, which invite professional lecturers from all walks of life to help build helpful learning environments. In conclusion, knowledge payment platforms have changed the traditional methods of acquiring knowledge by providing multiple courses to learners [11].

Information overload has generally been accepted as an issue since the digital technology became so indispensable [12]. As the amount of available information increases, individuals will likely become increasingly overwhelmed, which tends to hinder learning and innovation, reduce productivity and performance, and even affect the decision-making and well-being of individuals [13]. In addition to developing knowledge and skills in higher education or vocational education, incumbent learners need to rely on the opportunities in self-study for personal improvement. Generally speaking, in the self-learning stage, learners are eager to learn and improve their professional abilities. If individuals cannot clearly identify their learning needs, they may purchase knowledge products out of impulse, in order to meet the current demand perception. However, due to this impulse, individuals often purchase excessive knowledge products online and induce information complexity, which cannot be applied to short-term learning. Such behavioral patterns are discouraged by the overload of knowledge payment. As discussed above, individuals’ different motivations and personal background factors can affect decisions about future knowledge consumption behaviors [14].

As the knowledge economy develops, information sharing has extended from tangible assets to knowledge-based services [15]. In essence, knowledge payments are now being disseminated via fee-based services. The purpose of this study is to investigate whether the impact of certain factors on knowledge purchase intentions, in the context of knowledge consumption behavior, is influenced by personal motivation, involvement, information anxiety, or cognitive style. More explicitly, this study analyzes whether individuals over 20 years old prefer to use knowledge payment platforms (i.e., are knowledge demanders). There are two research objectives: (1) The impact of certain factors on learners’ knowledge purchase intentions; and (2) how learners make decisions to purchase online training course under the circumstances of overwhelming free online content.

## 2. Theoretical Background and Hypothesis Development

### 2.1. Relation of the Three Needs to Involvement

Khurana and Joshi [16] study an individual’s needs and suggest that one’s motivations can be predicted and learned based on his or her unsatisfied needs and feelings of anxiety. McClelland [17] three needs theory has been regarded as a basic factor in learning motivations. First, the need for achievement (nAch) refers to the defined goal of an individual to achieve, in terms of competition, i.e., to constantly steer toward and attempt to achieve their goals [18]. As Schüler, Sheldon [19] state, individuals with a high level of nAch are more willing to execute their goals. Second, the need for power (nPow) is defined as the desire of individuals to influence and control the power of others [20]. If individuals with a high level of nPow have high self-control and complete their learning goals with confidence, they often wish to control others [21]. Last, the need for affiliation (nAff) means that individuals who worry about being disliked tend to expect to maintain a good relationship with others [22]. Leary, M Kelly [23] demonstrate how individuals with a high level of nAff will often seek out interactions with other people to build stronger relationships. Therefore, the three needs, i.e., the need for achievement (nAch), the need for power (nPow), and the need for affiliation (nAff), are internal factors in this study. To better measure the abovementioned three needs, we refer to Royle and Hall [24], Kwaku Duah and Opoku [25], and Leary, M Kelly [23] to develop the scale items.

Learners are willing to apply their best and full effort when they have the greatest needs because they believe that their learning content is valuable or interesting [26]. Involvement is defined as a type of psychological state [27] in which the level of involvement in purchasing products is subject to personal needs and values [28]. With a shift in a consumer’s needs and values, his or her level of product involvement changes [29] because consumer needs are always based on knowledge of the desired product, which informs buying decisions [30]. Consumers’ various phases of life are constantly affecting which products they require due to their shifting needs [31]. Consumer behavior studies show that highly involved people frequently spend a lot of time assessing the quality of their products to ensure that they conform to their preferences [32]. Whereas, people with low involvement are less willing to look for relevant information [33]. Moreover, people tend to spend additional time and effort searching for information before making a purchase of a high involvement product [34].

This leads to our first hypotheses:

**Hypothesis** **1a** **(H1a).** *The need for achievement has a significant influence on involvement.*

**Hypothesis** **1b** **(H1b).** *The need for power has a significant influence on involvement.*

**Hypothesis** **1c** **(H1c).** *The need for affiliation has a significant influence on involvement.*

### 2.2. Relationship of Cognitive Style to Involvement and Information Anxiety

Cognitive style (CS) is a way of perceiving practical problems and dealing with issues in the process of decision-making [35]. A good learning environment offers learners enrichment that helps them reach breakthrough experiences [36]. Compared to many other cognitive styles, Witkin’s field dependence-independence (FDI) theory has been researched empirically in the context of instructional-design effects and individual variations, finding different learning preferences [37] and analogical reasoning [38]. Field-independent (FI) learners have a high analytical ability and prefer to complete their work independently, while field-dependent (FD) learners are easily influenced by complex environments and cannot find accurate information by themselves [39]. Sözcü, İpek [40] observe that learners of the FI type are more interested in the environment of e-learning. Similarly, Simuth and Sarmany-Schuller [41] propose that learners’ cognitive style preferences can have an indirect impact on some e-learning activity preferences, and the satisfaction and effectiveness of online learning. Therefore, cognitive styles are viewed as innate, and there are no good or bad styles [42,43]. Therefore, in line with these recent studies, we consider the following hypothesis:

**Hypothesis** **2** **(H2).** *Learners’ different cognitive styles may have different effects on involvement.*

Information technology has generally become an important tool for learning. It has also improved the level of convenience for people in the process of seeking knowledge and gaining a better understanding of the information they are receiving. However, overwhelming free content online might be the case to incur information overload for learners. Williamson and Eaker [44] note that people not only have greater life demands and undertake increasingly complex missions as they age but are also forced to face changing information and information overload problems. There can be simply too much information to digest [45]; people who do not know how to deal with what they think they should understand may feel powerless or desperate and experience information anxiety [46]. The lower the learner’s sense of information anxiety, the higher their learning ability and willingness to accept more knowledge and challenges [47,48]. Razavi, Shahrabi [49] also proposed that cognitive preference is associated with sense of anxiety. As discussed, FI learners enjoy nonlinear approaches to exploring problems, while FD tend to rely on step-by-step guidance. Therefore, the following hypothesis is proposed:

**Hypothesis** **3** **(H3).** *Learners’ different cognitive styles may have different effects on information anxiety.*

### 2.3. Relation of Information Anxiety to Involvement and Knowledge Purchase Intention

Information anxiety concerns not only the quantity of information but also the reception, processing, and application of information, including sensations of feeling uneasy [50]. When individuals experience internal uneasiness in situations, their behavioral pattern and psychological status can be affected indirectly [51]. Different levels of involvement reflect differences in individual cognition and higher purchase intentions for highly involved participants, thereby indicating that a product has value and attractiveness [52]. Furthermore, Bauer, Stokburger-Sauer [53] showed that the level of involvement can be understood in terms of cognitive value for individual products. In other words, the potential cognition of consumers indirectly determines consumers’ level of involvement with a product.

However, task-driven behavior is a key feature of knowledge consumption behavior [54], based on extrinsic factors and intrinsic emotions, which control the individual’s purchase intention through a platform to satisfy a need [55]. Additionally, Qi and Wang [56] suggest that a knowledge demander may not be able to identify the quality of knowledge products before making a purchase to ensure that the products match his or her needs. The above discussions indicate that people’s underlying psychographic characteristics and knowledge content values are affected when learners are willing to pay for knowledge [57]. These observations lead to the following hypotheses:

**Hypothesis** **4** **(H4).** *Involvement has a significant influence on knowledge purchase intention.*

**Hypothesis** **5** **(H5).** *Information anxiety has a moderating effect between involvement and knowledge purchase intention.*

### 2.4. Relation of Different Learners to Demand and Knowledge Purchase Intention

Associated with contemporary information technologies, consumers’ values, intentions, and corresponding behaviors are inferred by generational differences. Generation X (Gen X) is one of the most highly educated generations in history, and is characterized by technological and media savviness. Based on individuals’ particular socio-economic status, Gen X tends to focus on their potential for education and training, in order to confront and resolve their daily issues. Generation Y (Gen Y) pays more attention to the design, variety, ambience, layout, and their involvement in the shopping process [58]. Generation Z (Gen Z) is known as the mobile generation [59], and has a matured ability in solving problems through their communication and collaboration with their peers and family [60,61], and in their purchase behaviors [62]. Su, Li [63] indicate that gender has a significant influence on knowledge purchase intention. According to Punj [64], age and gender are related to knowledge consumption behavior; that is, females are more willing to pay than males, and younger consumers are more likely to be willing to pay for online content (e.g., music streaming services, etc.) as they are more familiar with online products. Based on these insights, we formulate the following hypotheses:

**Hypothesis** **6a** **(H6a).** *Learners of different ages show significant differences in the purchase intentions for knowledge-based products.*

**Hypothesis** **6b** **(H6b).** *Learners of different genders show significant differences in the purchase intentions for knowledge-based products.*

**Figure 2:**
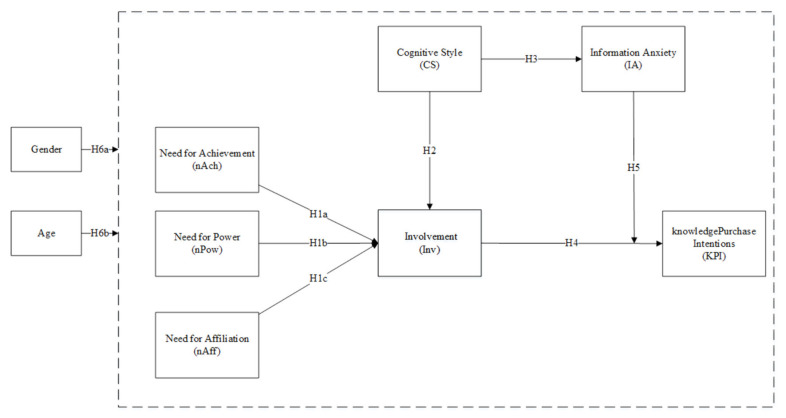
Research model of the demand to knowledge payment intention.

As stated above, the research models in Figure 2 are composed of seven theoretically well-grounded variables.

## 3. Methodology

In this study, the research method was developed to validate the relationship between learners’ demands and their purchase intentions for knowledge-based products.

### 3.1. Sample Procedure

According to the Taiwan Network Information Center [10] report, most e-learning users are aged 20 to 24. Consequently, this study focuses on incumbents over the age of 20 who prefer to use knowledge payment platforms. Therefore, this research classifies the research participants by age according to the XYZ generation of Lewis [65] and Kar [66]. Generation X (Gen X) refers to individuals 41–50 years of age and above, Generation Y (Gen Y) individuals are 31–40 years old, and Generation Z (Gen Z) refers to individuals 20–30 years old.

### 3.2. Measure

The constructs of this study were measured using 7-point Likert scales modified from previous literature. The complete measures include four dimensions: (a) Demographic and screen questions; (b) individual style; (c) personal perceptions; and (d) personal intentions. A questionnaire was developed with 36 items. Part 1 consisted of screening questions. Sequential screening was used for age (over 20 years old), work type (full or part-time), and experience using the knowledge products. If the participants did not meet all three conditions, they were not in consonance with this study. Table 1 lists the operational definition of all the dimensions. The complete scale is presented in Appendix A.

### 3.3. Post Hoc Testing for Common Method Variance (CMV)

To examine the common methods variance (CMV), we adopted the Harman’s one-factor test, according to Podsakoff, MacKenzie [67]. The first factor accounted for 35.77% of the variance, and all factors accounted for a total of 62.41% of the variance. That is, one single factor did not account for the majority of the covariance among the measures. Therefore, the CMV was not an issue as we report the finding by using the self-reported measures.

## 4. Results and Discussion

### 4.1. Descriptive Analysis

The study adopted purposeful sampling to invite incumbents over 20 years old who, as screening conditions, could provide accounts of knowledge product platforms and purchased knowledge-type course products. Out of 581 returns, 175 responses were removed as the participants had no experience or intentions in purchasing knowledge products. A total of 406 valid responses were entered to the data analysis. The results showed that there were more females (*n* = 228, 71%) than males (*n* = 118, 29%). Most of the participants were Gen Z between 20 and 30 years old (*n* = 318, 78.3%). Their work types were mainly part-time (*n* = 248, 61.1%) as shown in Table 2.

To avoid the social desirability when using self-reports, Podsakoff and Organ [68] suggest that researchers should identify whether the possible common method variance (CMV) occurs and influences relationships among the measures. One of the most widely used techniques that has been used by researchers to address the issue of CMV is Harman’s single-factor test [67]. The Harman’s single-factor test examines whether a substantial amount of either a single factor will emerge from the factor analysis or one general factor will account for the majority of the covariance among the measures [67]. Our study shows that 25.86% of the variance account for the majority of the covariance among the measures, which represents a statistically acceptable level of CMV.

### 4.2. The Measurement Model

Table 3 shows the results of the reliability and convergent validity of the constructs examined in this study. All the constructs’ CR values are between 0.78 and 0.926 (>0.7), indicating good internal consistency. The average variance extracted (AVE) values of the constructs also exceed 0.5 (from 0.518 to 0.751), which indicates the satisfactory convergent validity of the constructs [69,70]. Fornell and Larcker [69] suggest that AVEs’ square root (diagonal values) is higher than the intercorrelation among constructs. As shown in Table 4, we consider the model to have discriminant validity.

### 4.3. The Structural Model

**Figure 3:**
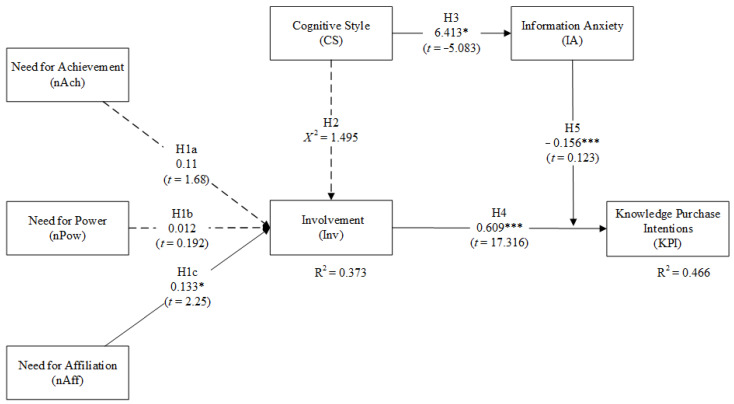
The test results of the research model. Note: * *p* < 0.05, *** *p* < 0.001. R^2^ refers to determination coefficient; χ^2^ refers to distribution coefficient; *t* value refers to coefficient of Student’s *t*-test.

As shown in Figure 3, as determined with bootstrap resampling methods, the determination coefficient R^2^ for Inv is 0.373 and 0.466 for KPI, which indicates that the measures explained 37.3% to 46.6% of the variance. From the results of a structural model inspection, only three hypotheses, namely, H1a, H1b and H2, are not statistically significant (*p* > 0.05), while the rest are indicated to be statistically significant. The empirical results show that (1) learners’ Inv is significantly associated with nAff (β = 0.133, *t* = 2.271, *p* < 0.001) but is not significantly associated with nAch (β = 0.11, *t* = 1.68, *p* > 0.05) or nPow (β = 0.012, *t* = 0.192, *p* > 0.05); (2) learners’ KPI is significantly associated with Inv (β = 0.609, *t* = 17.316, *p* < 0.001); and (3) IA (β = −0.156, *t* = 4.123, *p* < 0.001) was assessed as a moderating variable in the relationship between Inv and KPI. The empirical results support Hypotheses H1c, H4 and H5.

### 4.4. Statistical Analysis

We compared the different cognitive styles (CS) and involvement (Inv) groups of learners. First, CS and Inv were categorical variables. The different CS of learners were field-dependent (FD) (*n* = 194, 47.8%) and field-independent (FI) (*n* = 212, 52.2%). Second, Inv was assessed by quartile deviation and divided into three levels: Low (25th percentile, *n* = 113), medium (50th percentile, *n* = 176), and high (75th percentile, *n* = 117).

#### 4.4.1. Chi-Square Test

A Pearson chi-square test was used to test whether there are statistically significant differences influencing learners’ involvement between cognitive style, gender, Gens, and work type (Table 5). An association between CS and Inv was not observed, χ^2^(2) = 1.495, *p* > 0.05, which indicated that CS is not statistically significant in explaining Inv; consequently, H2 is not confirmed.

Additionally, regarding the effects of different background variables on Inv, the results show a striking effect of learners’ Gens (ages) on Inv in our test, χ^2^(4) = 11.88, *p* < 0.05, which indicates that involvement levels vary according to learners’ Gens. The lowest levels of involvement mainly occurs in GenY, while a level of medium involvement is in GenX; the highest involvement level is in GenY.

#### 4.4.2. Independent Sample *t*-Test

As Table 6 shows, the results suggest that FD with a mean of 4.56 has a significantly higher level of IA than an FI with a mean of 3.9 (*t* = −5.083, *p* < 0.05). This means that FD learners experience information anxiety more easily than FI learners. This result supports that learners’ different CS affect IA in this study; thus, H3 is confirmed.

#### 4.4.3. Three-Way ANOVA

A three-way ANOVA was used to determine the main and interactive effects on the mean number of learners’ Gens, genders and CS in relation to involvement and IA.

A significant effect of the condition was observed for Gens, *F* (2, 394) = 5.299, *p* < 0.05 partial η^2^ = 0.026, and gender, *F* (1, 394) = 4.9, *p* < 0.05, partial η^2^ = 0.012, and a significant Gens * gender relationship was observed, *F*(2, 394) = 5.059, *p* < 0.05, partial η^2^ = 0.025 (Table 7). Post hoc tests revealed that significant differences were observed in the males, *F* (2, 117) = 7.046, *p* < 0.05, and the responses of the GenY age group (*M* = 5.79, *SD* = 0.77) had more involvement than those of the GenZ age group (*M* = 5.21, *SD* = 0.91). Additionally, in the GenY age group, the gender difference had a significant difference in involvement level, *F* (1, 26) = 9.403, *p* < 0.01, and the male responses (*M* = 5.79, *SD* = 0.765) had more involvement than the female responses (*M* = 5.04, *SD* = 1.01) (Table 8).

Table 9 shows that the interaction effects are not statistically significant, but the main effect of factor A (CS) is statistically significant, *F* (1, 394) = 5.878, *p* < 0.05, partial η^2^ = 0.015. The means indicate that the comparison with FI learners (*M* = 3.92, *SD* = 1.37) and FD learners (*M* = 4.56, *SD* = 1.18) tends to have more information anxiety.

#### 4.4.4. Multi-Group Analysis (MGA)

To conduct group comparisons, the structural model uses the multi-group approach (PLS-MGA) of Henseler, Ringle [71]. As indicated in Table 10, the findings of this study support significant differences in learners’ genders in regard to the effect of IA mediating the relationship between Inv and KPI; thus, H6a is partially substantiated. As indicated in Table 11, no statistically significant differences are found in the three age groups; thus, H6b is not confirmed.

The purpose of this research was to examine learners’ relationships between basic demands and knowledge purchase intentions. The empirical findings suggest that learners’ nAff make learners more willing to pay to obtain knowledge, as they often have common topics to discuss with others. Therefore, H1c receives support. The results of the study show that learners’ nAch and nPow have no significant impact on a knowledge product’s level of involvement. Despite the appropriate learning environment, a learners’ nAch may not necessarily be impacted if a task demand is unclear [72]. Moore, Grabsch [73] show that learners’ nPow is not as important in the learning stage; learners are more focused on how to be leaders. Therefore, H1a and H1b are not supported. Furthermore, it was found that age does not interfere with each path in the multi-group analysis; thus, H6b is not supported.

Cognitive style is inborn and determines a person’s preferred way of thinking [43]. This study expected that learners’ different cognitive styles would affect their level of involvement in knowledge products; however, the findings show that H2 is not supported. Learners’ level of involvement in knowledge products is not affected by their cognitive styles (FI or FD). In terms of learners’ information anxiety, empirical research has shown that different cognitive styles can affect learning in different ways, especially for FD learners, who experience information anxiety more easily than FI learners. Therefore, H3 is supported.

As with all prior purchase considerations, learners’ demand determines the level of involvement for a knowledge product. Our results show that the effects of the H4 path are strongest when compared to other paths. This finding is consistent with Zhao, Zhou [15], which indicates that obtaining complete product information from potential suppliers ensures that knowledge products from trustworthy knowledge suppliers match learners’ needs. In terms of learners’ age, the level of high involvement is distributed among 31–40 year olds, as they attach great importance to knowledge-based products and believe that these can create value for them. In this sense, this paper supports the view that the level of involvement is a key factor in future purchase intention.

Additionally, the effects of interference definitively indicate a negative interaction between information anxiety and involvement on knowledge purchase intentions; thus, H5 is supported. When learners have high information anxiety, their cognitive abilities in knowledge products can be affected, which may lead to reduced purchase intentions in the future. In addition, the PLS-MGA results show that there are significant gender differences on this path. This demonstrates that information anxiety mostly stems from learners’ ability to receive and process information. Nevertheless, age groups are nonsignificant on all paths, which means that age is not the main factor in the effect of knowledge purchase intentions.

Finally, different genders and ages have interactive effects on involvement. In the simple main effect test, GenY learners’ involvement levels were higher than those of GenZ learners. Additionally, the main effects of cognitive style and information anxiety were significant, and FD learners’ information anxiety was higher than FI learners’, which is consistent with results from Chen [74].

## 5. Conclusions and Future Research

Our study’s framework surrounds the relevant factors that determine incumbent learners’ basic demands for knowledge-based products and focuses on learners’ knowledge consumption behavior. The study allowed us to reach the following four conclusions:

First, we adopted a three needs theory with a broader view toward learners’ knowledge consumption behaviors. The results showed that learners’ nAff will make them more willing to pay for intangible knowledge. The results also suggest that knowledge-based products can establish common topics and interpersonal relations in learning.

Second, this study investigated purchase intentions via online knowledge-based service platforms. Therefore, using cognitive styles to examine the characteristics of learners’ different cognitive behaviors, the results showed that regardless of which cognitive style is used by learners, their cognitive abilities will not be affected by knowledge-based products. This is an important finding for knowledge suppliers and platform providers. It suggests that brand positioning should be done properly to avoid homogenization, i.e., implementing brand value as a method to attract demanders should be considered a form of marketing strategy.

Third, although knowledge payment platforms can help learners quickly gain knowledge, the use of knowledge-based products is a pain point for learners. Therefore, information anxiety as a moderating variable was used to determine whether learners’ anxiety affects the relationship between knowledge-based product involvement and purchase intentions. The results showed that FD learners have more information anxiety than FI learners on knowledge-based product involvement and purchase intention. Consequently, the exchange activities provided by knowledge payment platforms tend not to help FD learners during their studies. Platform providers should propose solutions to relieve the pain points arising from demands and stable qualities for learning contexts.

Fourth, involvement is the key factor affecting purchase intentions in the future. We showed that the interaction between gender and age influences involvement, especially among 31 to 40-year-old male learners (Generation Y). Knowledge payment platforms can transform fragmented knowledge into systematic learning; for example, through a shortened version of a learning plan. Male learners 31–40 years of age (Generation Y) have specific knowledge needs, and typically consider knowledge-based products can increase their professional knowledge and skills to improve job opportunities.

As discussed above, this study’s hypotheses together frame our overall model for demonstrating the impact of learners’ basic demands on knowledge-based product purchase intentions. In the future, research directions and suggestions should be provided to knowledge payment platform operators as the bases of their decision-making. First, knowledge payment entails self-education, and this study is a pilot study; thus, some background factors of learners have not been included, such as learners’ salaries or their preferred prices for purchasing knowledge-based products. Moreover, it is recommended that further research should focus on confirming whether the higher involvement of 31 to 40-year-old male learners is related to job performance or professional fields. Given the different cognitive styles of learners, it is recommended that future researchers also investigate trust and satisfaction across platforms to identify whether external factors influence learning processes.

In conclusion, three elements of knowledge payment can further knowledge payment development, especially for knowledge platforms that aim to output and input knowledge-based products for revenue. Therefore, creating the core value of a brand is key, as it makes learners aware of its advantages. Brand positioning helps establish connectivity with a product and executes knowledge consumption behavior. Overall, we suggest that future research should focus on brand image and knowledge-based product marketing strategies.

## Figures and Tables

**Table 1:** The operational definition of all dimensions.

Dimension	Variable	Operational Definition	Items
Demographic and screening	demographic information	Demographic information including gender, age, work type, and most importantly, the experience of purchasing knowledge products as the screening item.	3
Individual style	three needs	nAch refers to an individual’s potential to challenge new goals during learning. nPow refers to an individual’s desire to influence or control others during the learning phase. nAff refers to an individual’s preference for communicating with people while learning.	12
Personal perceptions	Inv	Learners’ degree of perceived importance and cognitive value of knowledge-based products.	6
	CS	FI learners are used to setting goals by themselves. FD learners are used to communicating with people and accomplishing goals together during the learning phase.	6
	IA	Learners’ sense of information overload during the learning phase.	5
Personal intentions	KPI	Learners’ knowledge purchase willingness and the possibility for learning intention.	4

Note: nAch = needs for attachment; nPow = need for power; nAff = needs for affiliation; Inv = involvement; CS = cognitive style; IA = information anxiety; KPI = knowledge purchase intention.

**Table 2:** Demographic statistics.

Demographic Profile		*n*	%
Gender	Male	118	29%
	Female	288	71%
Age (years)	Gen Z (20–30)	318	78.3%
	Gen Y (31–40)	61	15%
	Gen X (41–55 and above)	27	6.7%
Work type	Full-time	158	38.9%
	Part-time	248	61.1%

**Table 3:** Results of convergent validity and reliability analysis.

Constructs	CR	AVE
FD	0.78	0.542
FI	0.881	0.711
nAch	0.858	0.604
nPow	0.829	0.549
Inv	0.926	0.677
KPI	0.923	0.751
nAff	0.809	0.518
IA	0.903	0.651

**Table 4:** The discriminant validity.

Constructs	FD	FI	nAch	nPow	Inv	KPI	nAff	IA
FD	**0.736**							
FI	0.41	**0.843**						
nAch	0.34	0.534	**0.777**					
nPow	0.314	0.351	0.552	**0.741**				
Inv	0.523	0.457	0.391	0.322	**0.823**			
KPI	0.432	0.445	0.341	0.289	0.671	**0.866**		
nAff	0.422	0.267	0.322	0.5	0.375	0.368	**0.72**	
IA	0.226	0.002	−0.045	0.179	0.22	0.269	0.271	**0.807**

Note: diagonal values refer to the square root of AVE. Off-diagonal elements represent correlations between the constructs.

**Table 5:** Results of the chi-square test.

Variables	Value	df	Asymptotic Significance (2-Tailed)
CS	1.495	2	0.474
Gender	2.111	2	0.348
Gens	11.88	4	0.018 *
Work type	2.614	2	0.271

* *p* < 0.05.

**Table 6:** Independent Sample *t*-test for different CS.

	**Mean (SD)**		***t*-Test**
	**FI**	**FD**	
IA	3.9 (1.37)	4.56 (1.18)	−5.083 *

* *p* < 0.05.

**Table 7:** Three-way ANOVA for involvement (Inv).

Source	df	SS	MS	*F*
CS (A)	1	0.219	0.219	0.205
Gen (B)	2	11.336	5.668	5.299 **
Gender (C)	1	5.241	5.241	4.9 *
A × B	2	2.095	1.048	0.979
A × C	1	0.137	0.137	0.128
B × C	2	10.822	5.411	5.059 **
A × B × C	2	5.876	2.938	2.747
Error	394			

* *p* < 0.05, ** *p* < 0.01.

**Table 8:** Simple main effects of gens and gender for involvement (Inv).

Cluster	*df*	SS	MS	*F*	Post Hoc
Gens					
Male	2	12.95	6.475	7.046 *	GenY > GenZ
Female	2	0.479	1.142	0.210	
Gender					
GenZ 20–30	1	0.11	0.11	0.096	
GenY 31–40	1	8.4	8.4	9.403 **	male > female
GenX 41–50 and above	1	0.627	0.627	0.923	

* *p* < 0.05, ** *p* < 0.01.

**Table 9:** Three-way ANOVA for information anxiety (IA).

Source	*df*	SS	MS	*F*
CS (A)	1	9.661	9.661	5.878 *
Gens (B)	2	6.321	3.161	1.923
Gender (C)	1	0.052	0.052	0.032
A × B	2	0.574	0.287	0.175
A × C	1	0.071	0.071	0.043
B × C	2	1.453	0.726	0.442
A × B × C	2	2.556	1.278	0.778
Error	394			

* *p* < 0.05.

**Table 10:** Assessment of group difference for gender.

Hypotheses	Relationship	*p*-Values Female vs. Male	Results
H1a	nAch→Inv	0.498	Unsupported
H1b	nPow→Inv	0.664	Unsupported
H1c	nAff→Inv	0.409	Unsupported
H4	Inv→KPI	0.517	Unsupported
H5	Inv * IA→KPI	0.014 *	Supported

* *p* < 0.05.

**Table 11:** Assessment of group difference for age.

Hypotheses	Relationship	*p*-Values		
		GenZ vs. GenY	GenY vs. GenX	GenZ vs. GenX
H1a	nAch→Inv	0.582	0.534	0.721
H1b	nPow→Inv	0.099	0.061	0.205
H1c	nAff→Inv	0.18	0.321	0.606
H4	Inv→KPI	0.502	0.186	0.266
H5	Inv * IA→KPI	0.435	0.964	0.456

* *p* < 0.05.

## Data Availability

The data that support the findings of this study are available from the corresponding author, upon request.

## Appendix A

**Table A1:** Measurement Scale Items.

Construct		Item
Three needs	nAch_1	Compared to accidental success, I prefer to overcome all difficulties with my ability.
	nAch_2	I am confident to complete the task.
	nAch_3	I like to challenge difficult tasks.
	nAch_4	I am satisfied with my abilities.
	nPow_1	I am afraid of being compared by others.
	nPow_2	I long to have the power to control others.
	nPow_3	I have the ability to influence others.
	nPow_4	I expect to get a good reputation.
	nAff_1	I tend to cater to others
	nAff_2	When I am not in the plan of others, I would feel disappointed.
	nAff_3	I look forward to keeping in touch with others.
	nAff_4	I would love to meet other people.
Involvement	Inv_1	I am interested in knowledge products.
	Inv_2	Knowledge products are indispensable.
	Inv_3	I love knowledge products.
	Inv_4	I feel fun when buying knowledge products.
	Inv_5	Knowledge products can show my personal characteristics.
	Inv_6	The knowledge products purchased can show personal interests and preferences.
Cognitive Style	FI_1	When learning new knowledge, I prefer to study alone.
	FI_2	I will set my own learning goals.
	FI_3	I can make good use of the resources around me.
	FD_1	When learning new knowledge, I prefer to be guided by others.
	FD_2	I expect learning suggestions based on my interests.
	FD_3	I am willing to participate in learning related activities.
Information Anxiety	IA_1	When looking for information, I feel anxious or frustrated.
	IA_2	When looking for information, I am worried that I cannot find the information I want.
	IA_3	I spend a lot of time searching for new information.
	IA_4	I feel uneasy when I learn new knowledge.
	IA_5	Faced with a lot of information, I feel anxious.
Knowledge Purchase Intention	KPI_1	I recommend to others to buy knowledge content products.
	KPI_2	I continue to buy knowledge products.
	KPI_3	I acquire more knowledge through the knowledge platform.
	KPI_4	Buying knowledge content products is valuable.
